# Preventive Effect of Flavonol Derivatives Abundant Sanglan Tea on Long-Term High-Fat-Diet-Induced Obesity Complications in C57BL/6 Mice

**DOI:** 10.3390/nu10091276

**Published:** 2018-09-10

**Authors:** Ponmari Guruvaiah, Huimin Guo, Daxiang Li, Zhongwen Xie

**Affiliations:** State Key Laboratory of Tea Plant Biology and Utilization, School of Tea and Food Sciences and Technology, Anhui Agricultural University, Hefei 230036, China; ponmariguruvaiah@gmail.com (P.G.); guohuimin2008@hotmail.com (H.G.); dxli@ahau.edu.cn (D.L.)

**Keywords:** sanglan tea, obesity, high-fat diet, lipogenesis, non-alcoholic fatty liver, chemical profile

## Abstract

Sanglan Tea (SLT) is a Chinese medicine-based formulation that is consumed as a health drink for the effective management of obesity-associated complications. However, its chemical components and mechanism of action in the prevention of hepatic steatosis and obesity-related impairments have been uncertain. In this study, we aimed to unveil the chemical profile of SLT and to explore its preventive mechanism in high-fat-diet-induced non-alcoholic fatty liver disease (NAFLD) and obesity-related consequences in C57BL/6 mice. Ultrahigh-performance liquid chromatography (UHPLC) coupled to a quadrupole-orbitrap high-resolution mass spectrometry (MS) analysis of SLT indicated that analogs of quercetin and kaempferol are major compounds of flavonoids in SLT. A dietary supplement of SLT efficiently managed the blood glucose elevation, retained the serum total cholesterol (TC), LDL-cholesterol (LDL-C), and triglyceride (TG) levels, as well as aspartate aminotransferase (AST) and alanine aminotransferase (ALT) activity, and reduced the fat storage in the liver induced by a high-fat diet. The underlying mechanism of this preventive effect is hypothesized to be related to the inhibition of over-expression of lipogenesis and adipogenesis-related genes. Overall, this study suggests that SLT, being rich in quercetin and kaempferol analogs, could be a potential food supplement for the prevention of high-fat-diet-induced NAFLD and obesity-related complications.

## 1. Introduction

Obesity is defined as excessive fat accumulation in adipose tissue, and arises when the intake of energy far exceeds the burning of calories, and the excess energy is stored, in the form of fat, as adipose tissue. Recently, the prevalence of obesity has increased drastically in both children and adults. Often, obesity is associated with several risk factors, such as insulin resistance, cardiovascular diseases, dyslipidemia, hypertension, and hepatic steatosis [1,2,3]. At present, a wide variety of drugs are being used for the treatment and prevention of obesity, particularly Orlistat, a semisynthetic and hydrogenated derivative of lipstatin that has been widely used in numerous in vitro studies. However, these pharmacological agents are limited in efficacy and have potential side-effects [4]. Therefore, an alternative therapy for obesity-related complications is needed.

Sanglan tea (SLT) is a Chinese medicinal formula consisting of *Gynostemma pentaphyllum*, *Morus alba*, and *Nelumbo nucifera*, with a ratio of 13:4:3. It has been used as a health drink in traditional Chinese medicine, as its ingredients prevent and treat various diseases—mainly obesity-associated complications [5,6,7].

Recently, pronounced antioxidant and agonistic ligand activity of flavonoid glycosides isolated from *G. pentaphyllum* aerial parts against the peroxisome proliferator-activated receptor, which is involved in hepatic adipogenesis, were reported [8,9]. During adipogenesis, pre-adipocytes increased in both number and size to become mature adipocytes, leading to an increase in fat mass. In some in vitro studies, herbal extract supplementation administered to high-fat diet-induced obese mice resulted in a significant decrease in adipogenic transcriptional factors such as peroxisome proliferator-activated receptor-gamma (PPAR-γ), CCAAT/enhancer binding protein-a (C/EBP-a), and sterol regulatory element-binding protein (SREBP-1c)-associated proteinss, including adipocyte protein 2 (aP2) and fatty acid synthase (FAS), implicating the process of adipogenesis [10]. Additionally, an extract and gypenoside XL from *G. pentaphyllum* alleviated fatty degeneration and hepatic fibrosis in mice with non-alcoholic fatty liver disease (NAFLD). The overexpression of acyl-CoA oxidase (ACO) and carnitine palmitoyltransferase-1 (CPT-1) may be involved in the hepatoprotective effects by regulating mitochondrial fatty acid β-oxidation [11]. Additionally, gypenosides isolated from *G. pentaphyllum* showed inhibitory effects on lung cancer cell growth [12] and improved diabetic cardiomyopathy [13]. A phytochemical and pharmacological study reported that kaempferol isolated from a water extract of *N. nucifera* leaves reduced the expression of different adipogenic transcription factors and their target genes [14]. In addition, nuciferine from *N. nucifera* leaf ameliorated hepatic steatosis in high-fat diet/streptozocin-induced diabetic mice [15]. It was reported that flavonoids from *Nelumbo nucifera* leaves, including quercetin, catechin, hyperoside, isoquercitrin, and astragalin, exhibit lipolytic activity and have hypolipidemic effects on adipose tissue [16,17]. Various studies have demonstrated that bioactive constituents such as isoquercitrin, chlorogenic, acid and rutin from *M. alba* (Mulberry) leaves ameliorate hyperglycemia in type 2 diabetes [18,19]. Jung et al. has demonstrated that quercetin supplementation, administered to high-fat-diet-induced obese mice, reduces fat storage and alters the transcription of lipogenesis-related genes in the liver [20]. Lipogenesis is a complex process that regulates the synthesis of endogenous lipids and unsaturated fatty acids, followed by their saturation and/or elongation in the liver and adipose tissue [21]. Wang et al. studied the effect of an herbal extract on lipogenic gene expression (e.g., fatty acid synthase (FAS), acetyl-CoA carboxylase (ACC), and stearoyl-CoA desaturase (SCD-1)), and a transcriptional factor involved in fatty acid esterification was controlled by a sterol regulatory element-binding protein (SREBP-1). It was suggested that the up-regulation of SREBP-l was suppressed by natural product supplementation in obese mice. An SREBP-1 level increase in a fatty liver, which further increases the expression of lipogenic genes, thereby enhances fatty acid synthesis and accelerates triglyceride accumulation [21]. 

Recently, scientific studies regarding the health promotion of natural resources largely focuses on the chemical constituents and explores the mechanism of metabolites in food supplements. Chemical constituents have always been the key elements in the pharmacological effects of food supplements. Previous reports suggest that herbs containing SLT are rich in phenolics, mainly flavonol derivatives [22,23,24].

The chemical profiling of SLT has not yet been analyzed. In addition, there are no scientific reports on the role of SLT against obesity complications, and its underlying molecular mechanism remains uncertain. Therefore, this study aimed to substantiate the key metabolites of SLT and hypothesize its possible underlying preventive mechanisms of action in high-fat-diet (HFD)-induced obesity and the associated non-alcoholic fatty liver in C57BL/6 mice.

## 2. Materials and Methods 

### 2.1. Ultrahigh-Performance Liquid Chromatography (UHPLC)-Orbitrap-MS Analysis of SLT

Sanglan Tea (SLT) was provided by Tiesanglan Biotech Inc.(Shangdong, China). Approximately 10 g of SLT powder was extracted with 200 mL pre-heated distilled water at 100 °C (at the ratio 1:20). Extraction was assisted by an ultrasonic (KQ-500DE Shumei, Kunshan, China) water bath for 30 min at 75 °C. After 50-fold dilution, samples were centrifuged at 15,000·rpm at 4 °C for 10 min, and then filtered through a 0.22 µm filter for chemical constituent analysis. Chromatographic analysis was performed on an Ultimate 3000 UHPLC system equipped with a Q Exactive Focus mass spectrometer (Thermo Fisher Scientific, Waltham, MA, USA). SLT was separated using a Hypersil GOLD column (I.D. 100 mm × 2.1 mm, 1.9 µm; Thermo Fisher Scientific, Waltham, MA, USA). The column temperature was held at 40 °C and the mobile phase was composed of A (0.1% formic acid in water) and B (acetonitrile containing 0.1% formic acid) at a flow rate of 0.3 mL/min under gradient elution conditions: 5–95% B at 0–12 min.

Mass spectrometer (MS) detection was performed on a Q Exactive Focus mass spectrometer (ThermoFisher Scientific, Waltham, MA, USA) equipped with ESI. Positive and negative ionization mode detection was acquired in a full scan operation, with a mass range of 70–1050 *m/z* using a spray voltage of 3.8 kV and 3.1 kV. Mass spectra and chromatograms were acquired and processed with XCalibur version 2.1 (ThermoFisher Scientific, Waltham, MA, USA). The accurate mass and composition of the precursor and fragment ions were processed and sequenced using the Compound Discoverer version 2.0 (ThermoFisher Scientific, Waltham, MA, USA). The stability of the method was tested by performing a quality control of every six samples.

### 2.2. Preparation of Water Extract of SLT 

Water extracts of SLT were prepared following previous methods, with slight modifications [25]. SLT powder was extracted with pre-heated distilled water at 100 °C (the ratio of SLT powder to pure water was 1:20). Extraction was assisted by an ultrasonic (KQ-500DE Shumei, Kunshan, China) water bath for 30 min at 75 °C. The resulting extract was filtered and evaporated in a rotary evaporator under vacuum at 25 °C (IKA^®^ HB 10, Staufen, Germany). The fine powder was stored at −20 °C until use.

### 2.3. Experimental Animals and Treatment

Briefly, sixty 5-week-old C57BL/6 male mice were purchased from the National Resource Centre of Model Mice (NRCMM, Nanjing, China) and were allowed to acclimate to the environment for one week at the specific pathogen-free (SPF) animal facility at Anhui Agricultural University. Mice were housed in cages and allowed free access to the water and standard chow diet for a week in a room maintained at 22 ± 1 °C with 50 ± 5% relative humidity and a 12 h light/dark cycle.

At 6 weeks of age, the mice were divided into either normal chow diet (ND, *n* = 12) or high fat diet (HFD, *n* = 48) groups, which were fed ND or HFD, respectively, for 28 weeks. HFD mice were further assigned to four subgroups. The first group of mice (*n* = 12) received a high fat diet only, the second and third group of mice (*n* = 12 each) were fed HFD and received intragastric supplements of SLT every day at a dosage of 200 and 400 mg/kg body weight (BW), respectively (SLT-200 and SLT-400), and the fourth group of mice (*n* = 12) received HFD while simultaneously being intragastrically administered 10 mg/kg BW of Orlistat daily (OR, Sigma Aldrich, St. Louis, MO, USA). The ND or HFD group mice were also intragastrically administered sterilized water at 10 mL/kg BW, respectively. All mice were fed ad libitum and treated for 28 weeks until they were sacrificed. The normal chow diet and high fat diet were obtained from Trophic Animal Feed High-Tech Co., Ltd. (Nantong, China).

Food intake and water consumption were monitored daily. Body weight was obtained with a weight scale weekly. Fasting blood glucose was recorded every week using a Nova StatStrip Xpresst^TM^ Glucose CR Meter (Nova Biomedical, Waltham, UK), with Nova StatStrip Xpresst^TM^ Glu-test Strips (Nova Biomedical, Waltham, UK). All the animal experimental procedures were in accordance with the guidelines of the institutional animal care and use committee (IACUC) of Anhui Agricultural University.

#### 2.3.1. Glucose and Insulin Tolerance Test 

A glucose tolerance test (GTT) was performed after 26 weeks of diet and treatment. The fasting mice were given an intraperitoneal injection of glucose (d-(+)-Glucose, Sigma Aldrich, St. Louis, MO, USA) at a dosage of 1.5 g/kg BW. The blood samples were collected from the tail veins of the mice, and glucose levels were measured at 0, 30, 60, 90, and 120 min after injection [26]. During an insulin tolerance test (ITT), animals that had fasted for 4 h were given an intraperitoneal injection (1 U/kg BW) of insulin (400 U/100 mL Wanbang, Jiangsu, China), followed by blood sample collections from tail veins after 0, 30, 60, 90, and 120 min. The glucose level was measured with Nova StatStrip Xpresst^TM^ Glu-test Strips [27].

#### 2.3.2. Collection of Serum and Tissue Samples

At the end of 28 weeks of treatment, animals were anesthetized by pentobarbital sodium injection (50 mg/kg, i.p.) upon overnight fasting and were sacrificed after peripheral blood collection from the ophthalmic vein. A serum was obtained by centrifugation at 3000 rpm/min for 5 min at 4 °C, then stored at −80 °C. Liver and subcutaneous fat were carefully removed and weighted on a scale. All other tissue samples were harvested and properly stored for further biochemical, molecular, and immunostaining analyses.

### 2.4. Biochemical Analysis

The levels of triglyceride (TG), low-density lipoprotein cholesterol (LDL-C), high-density lipoprotein cholesterol (HDL-C), total cholesterol (TC), aspartate aminotransferase (AST), and alanine aminotransferase (ALT) in the serum were measured using micro test kits from Nanjing Jiancheng Bioengineering Institute (Nanjing, Jiangsu, China). 

### 2.5. Hematoxylin–Eosin (HE) Staining

All the liver tissues fixed in 10% neutrally buffered formalin solution were dehydrated and embedded in paraffin (Paraplast Tissue Embedding Medium, LEICA, Buffalo Grove, IL, USA) using a modular tissue embedding system (LEICA EG1150 H, Buffalo Grove, IL, USA). The embedded tissues were cut into 5 μm sections using a fully automated rotary microtome (LEICA RM2255, Nussloch, Germany), and mounted onto positively charged slides (CITOGLAS, Shanghai, China). HE staining was performed using a HE staining kit (Boster Biological Technology Company, Pleasanton, California, USA) and was imaged by microscope (LEICA DM500, Wetzlar, Germany) using a supporting camera (LEICA ICC50 W, Wetzlar, Germany). The hepatic adipose infiltration cells were counted manually using Image J software (National Institutes of Health, Bethesda, MD, USA).

### 2.6. Real-Time PCR

Total RNA was extracted from liver tissues using an RNA isolator (Vazyme Biotech Co., Ltd., Nanjing, Jiangsu, China) in accordance with the manufacturer’s instructions. Reverse transcription was performed using a HiScript^®^ II 1st Strand cDNA Synthesis kit (Vazyme Biotech Co., Ltd., Nanjing, China). Real-time PCR was performed using a Bio-Rad CFX System and AceQ qPCR SYBR Green Master Mix kit (Vazyme Biotech Co., Ltd., Nanjing, China) following the method described previously [28,29]. The primer sequences listed in Table 1 are designed for mice genes.

### 2.7. Western Blot Analysis

Western blot was performed following the method described previously [29,30,31]. An equal amount of denatured proteins was separated by SDS-PAGE gels and transferred to nitrocellulose membranes. The membranes were blocked with 5% skimmed milk powder in a PBST solution for 1 h, and were incubated with peroxisome proliferator-activated receptor-γ (PPAR-γ) (Santa Cruz Biotechnology, CA, USA), sterol regulatory element-binding protein-1 (SREBP-1) (Santa Cruz Biotechnology, CA, USA), stearoyl-CoA desaturase -1 (SCD-1) (Cell Signaling Technology, MA, USA) and β-Actin (Proteintech^TM^, Wuhan, Hubei, China) at 4 °C overnight, then incubated with appropriate secondary antibodies (Proteintech^TM^, Wuhan, Hubei, China) for 1 h at room temperature. Protein bands were detected by an enhanced-chemiluminescent (ECL) reagent (Vazyme Biotech Co., Ltd., Nanjing, China) and analyzed using a ChemicDoc^TM^ MP Imaging System (Bio-Rad, Hercules, CA, USA) with a supporting system (ImageLab., Bio-Rad, Hercules, CA, USA).

### 2.8. Statistical Analysis

The results were expressed as mean ± standard error of mean (SEM). Comparisons between the two groups were performed with an unpaired two-tailed Student’s *t*-test, and multiple group comparisons were performed by one-way ANOVA, followed by Tukey’s test. *p* < 0.05 was used to consider statistical significance.

## 3. Results

### 3.1. Chemical Profiling of SLT

In order to establish a chemical profile of SLT, the samples were analyzed by UHPLC-Q-Exactive Focus equipped with Compound Discoverer software (Thermo Fisher Scientific, USA). A total of 621 and 433 metabolites were detected in positive and negative ion modes, respectively. Total ion chromatograms (TICs) of positive and negative ion modes are provided in the Supplementary Material (Figures S1 and S2). The chemical compounds were screened based on the measurement of the accurate mass and retention time.

The structural characterization of the chemical compounds was confirmed based on a comparison of the accurate mass measurement, elemental composition assignment, and MS/MS spectrum interpretation of bioactive metabolites data of herbs that contain SLT. Further, integration and peak assignment was verified for both the most abundant precursor-ion and the fragment-ion of the chemical compound, and untargeted data processing was carried out using the databases HMDB (http://www.hmdb.ca/) [32], GNPS (The Global Natural Product Social Molecular Networking, gnps.ucsd.edu) [33] and TCMSP (Traditional Chinese Medicine Systems Pharmacology database and analysis platform, http://sm.nwsuaf.edu.cn/lsp/tcmsp.php.) [34]. 

Finally, 24 compounds were tentatively assigned, including 15 flavonoids and 6 alkaloids with hydroxy and amino acids. The retention time and mass spectrum data, along with peak assignments for the identified metabolites, are described in Table 2. 

### 3.2. Quercetin and Kaempferol Derivatives 

Metabolite identifications 2, 3, 4, 5, and 6 (Table 2) show that quasi-molecular ions of (M + H)^+^ *m/z* at 611.15, 613.54, 479.08, 465.10, and 303.18 share fragment ions at *m/z* 303. The MS/MS spectra of kaempferol derivatives, and metabolite identifications 21 and 24 (Table 2), showed that quasi-molecular ions of (M − H)^−^ *m/z* at 593.15 and 447.13 share fragment ions at *m/z* 284/285. The quercetin and kaempferol MS/MS spectra as well as the masses of the fragment ions agree very well with fragmentation data reported for *Capparis spinosa* L [35].

### 3.3. SLT Prevented Body Weight Gain and Fatty Liver in HFD-Induced C57BL/6 Mice 

**Figure 1:**
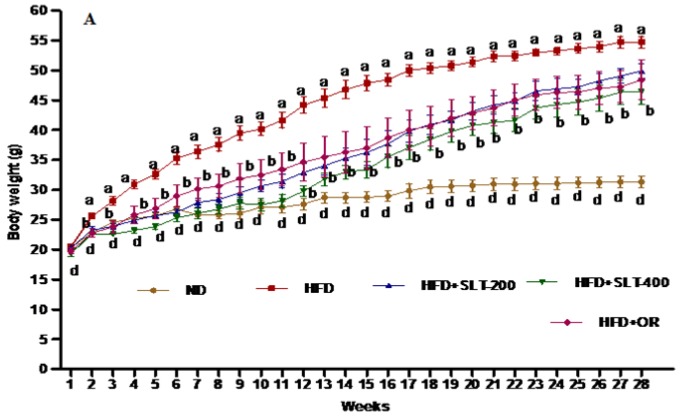
Sanglan tea (SLT) supplementation prevented a high-fat-diet (HFD)-induced body weight increase in C57BL/6 mice. Values with different letters are significantly (*p* < 0.05) different from each other. One-way analysis of variance (ANOVA), followed by Tukey’s test, were used to compare all pairs of columns. ND: normal diet; SLT-200, SLT-400: animals received intragastric supplements of SLT every day at a dosage of 200 and 400 mg/kg body weight (BW), respectively; OR: intragastric administration of Orlistat at 10 mg/kg BW/day.

The effect of SLT on the body weight of the mice fed with HFD for 28 weeks is shown in Figure 1. The body weight of mice fed HFD increased compared to mice fed ND. A significant difference in terms of the final body weight and the decrease in body weight gain was observed in HFD + SLT 200, HFD + SLT 400, and HFD + OR group mice when compared to mice fed HFD only (Table 3). At the same time, the liver weight index of HFD + SLT mice decreased significantly (*p* < 0.05) compared to the mice fed HFD only. Simultaneously, this long-term preventive study showed that there was no pronounced difference in the subcutaneous fat weight among any of the experimental groups at the end of the experiment. In terms of food intake, a significant variation was observed between mice fed ND compared to those fed HFD (Table 3). Thus, no difference in food intake was observed between mice supplemented with SLT and OR in comparison to those supplemented with HFD. Body weight gain was strongly suppressed by higher dose supplementation of SLT compared to mice fed HFD only.

### 3.4. SLT Prevented Serum Lipid Increase and Hepatic Abnormality in HFD Mice 

**Figure 2:**
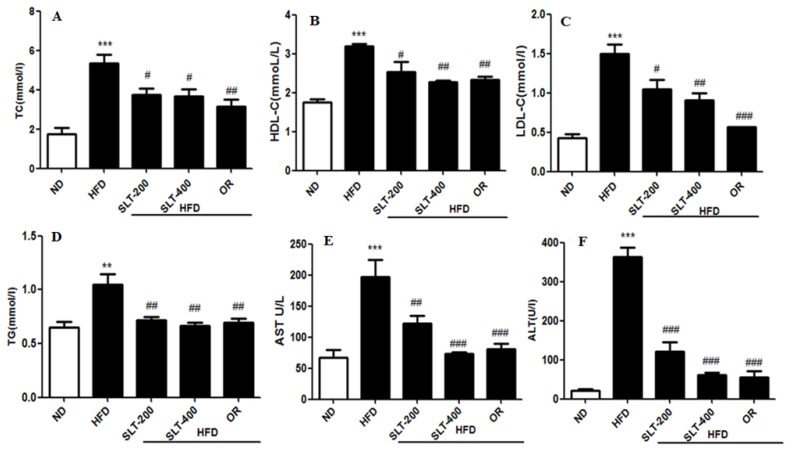
SLT supplementation retains serum biochemical parameters in C57BL/6 mice. (**A**) Total cholesterol (TC); (**B**) High-density lipoprotein cholesterol (HDL-C); (**C**) Low-density lipoprotein cholesterol (LDL-C); (**D**) Triglyceride (TG); (**E**) Aspartate aminotransferase (AST); and (**F**) Alanine transaminase (ALT). Values with different symbols are significantly (*p* < 0.05) different from each other. One-way analysis of variance (ANOVA), followed by Tukey’s test, were used to compare all pairs of columns. ** *p* < 0.01, and *** *p* < 0.001 versus ND, and ^#^ *p* < 0.05, ^##^ *p* < 0.01, ^###^ *p* < 0.001 versus HFD.

The serum lipid profiles of five groups of mice were shown in Figure 2. The mice fed the HFD had higher levels of TC (Figure 2A) and TG (Figure 2D) than mice fed the ND, whereas they were significantly ameliorated by 200 and 400 mg/kg BW SLT supplementation (*p* < 0.05). SLT administration decreased the LDL-C level (*p* < 0.05) in HFD mice, although there was no alteration in the ratio of LDL-C to HDL-C cholesterol. For evaluating the hepatic toxicity, the levels of the serum AST and ALT were also measured. Our data showed that SLT administration led to a dose-dependent decrease in the AST (Figure 2E) and ALT (Figure 2F) levels. Moreover, SLT administration showed itself to be as effective as positive OR treatments.

### 3.5. SLT Ameliorated Elevated Blood Glucose and Insulin Insensitivity in HFD Mice 

**Figure 3:**
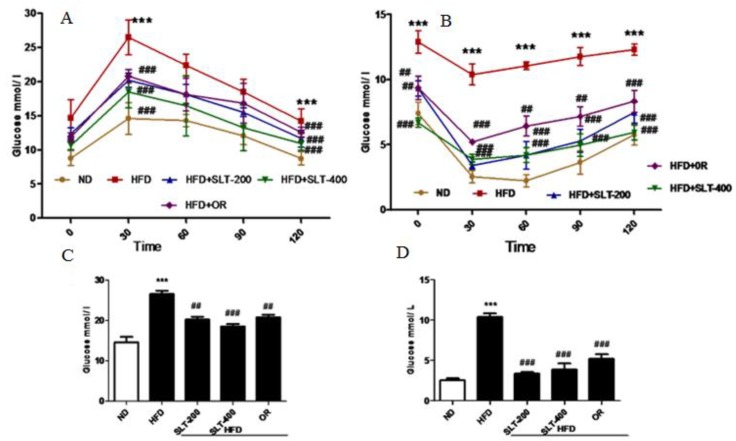
SLT supplementation efficiently ameliorated the elevated blood glucose in HFD C57BL/6 mice. (**A**,**C**) Oral glucose tolerance test (OGTT) and area under the curve (AUC) of OGTT; (**B**,**D**) Insulin tolerance test (ITT) and AUC of ITT. Values with different symbols are significantly (*p* < 0.05) different from each other. One-way analysis of variance (ANOVA), followed by Tukey’s test were used to compare all pairs of columns. *** *p* < 0.001 versus ND and ^##^ *p* < 0.01, ^###^ *p* < 0.001 versus HFD.

In addition to obesity phenotypes, the HFD mice also showed elevated fasting blood glucose levels. Therefore, the glucose tolerance test (GTT) and insulin tolerance test (ITT) were performed, and the results are shown in Figure 3. Mice fed HFD showed a marked increase of the glucose levels in serum at all time points (30, 60, 90, and 120 min) (Figure 3A). SLT supplementation and OR administration efficiently reduced the single-time-point serum glucose in the area under curve (AUC) level (Figure 3C). Meanwhile, HFD mice showed a significantly higher level of insulin compared to mice fed ND during the experiment. However, the serum glucose level was significantly attenuated in SLT-supplemented mice (Figure 3B). AUC values of ITT are also shown in Figure 3D. These data suggest that both dosages of SLT could efficiently improve glucose intolerance in HFD-induced mice. 

### 3.6. SLT Protected Fatty Liver Formation in HFD Mice

**Figure 4:**
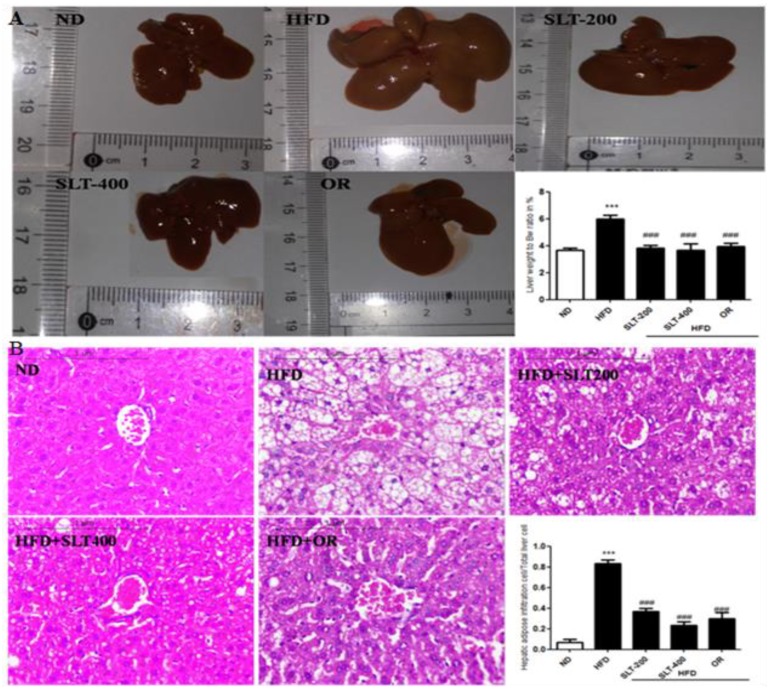
SLT supplementation preserved normal liver in HFD C57BL/6 mice. (**A**) Representative liver images and liver weight-to-BW ratio; (**B**) Representative images of liver tissue, Hematoxylin–Eosin (HE) staining, and quantification of fatty hepatic cells. Data are presented as mean ± SEM (*n* = 3–6). One-way analysis of variance (ANOVA) followed by Tukey’s test were used to compare all pairs of columns. ND: normal diet; HFD: high fat diet; SLT-200, SLT-400: animals received intragastric supplements of SLT every day at a dosage of 200 and 400 mg/kg body weight (BW), respectively; OR: intragastric administration of Orlistat at 10 mg/kg BW/day. *** *p* < 0.001 versus ND, and ^###^ *p* < 0.001 versus HFD.

Obesity is often associated with hepatic alterations. Our data show that HFD feeding resulted in increased serum AST and ALT levels (Figure 2E,F) in HFD mice. Our data also show that fat deposition in the liver was observed in both morphological (Figure 4A) and histological (Figure 4B) examination. Hematoxylin–eosin (HE) staining exhibited a substantial deposition of fat in the form of large vacuoles in the liver tissue of HFD mice, which is a state of a fatty liver. Conversely, SLT administration resulted in a greater reduction in the deposition of fat droplets. Furthermore, the supplementation of higher-dose SLT most effectively preserved normal live in HFD fed mice.

### 3.7. SLT Prevented Lipogensis and Adipogenesis in the Liver of HFD Mice

**Figure 5:**
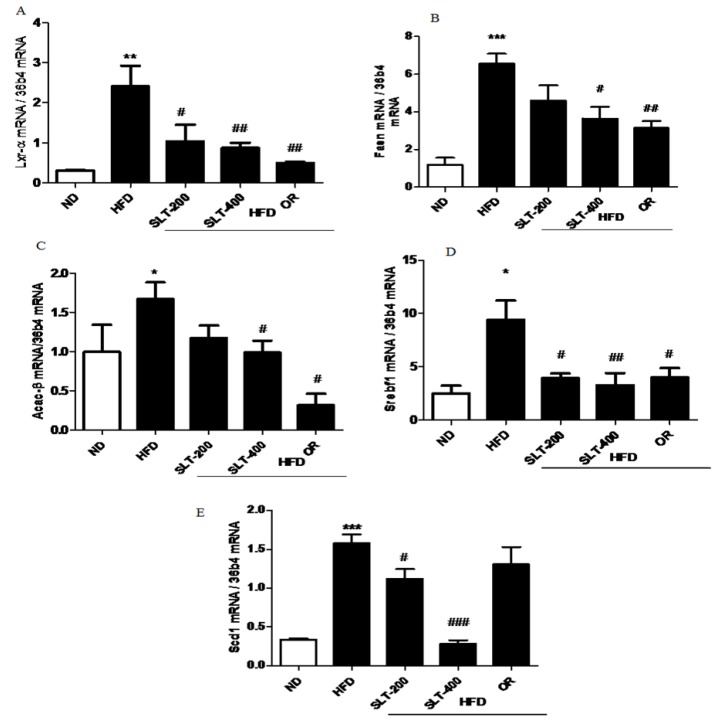
SLT supplementation decreased hepatic lipogenic gene expression in HFD mice. The mRNA expression of lipogenic genes (**A**) *Lxr-α*, (**B**) *Fasn*, (**C**) *Acac-β*, (**D**) *Srebf1*, and (**E**) *Scd1* in the liver tissue of HFD C57BL/6 mice. Values given are mean ± SEM (*n* = 3–6). One-way analysis of variance (ANOVA) followed by Tukey’s test were used to compare all pairs of columns. ND: normal diet; HFD: high fat diet; SLT-200, SLT-400: animals received intragastric supplements of SLT every day at a dosage of 200 and 400 mg/kg body weight (BW), respectively; OR: intragastric administration of Orlistat at 10 mg/kg BW/day. * *p* < 0.05, ** *p* < 0.01, and *** *p* < 0.001 versus ND; ^#^ *p* < 0.05, ^##^ *p* < 0.01, and ^###^ *p* < 0.001 versus HFD.

**Figure 6:**
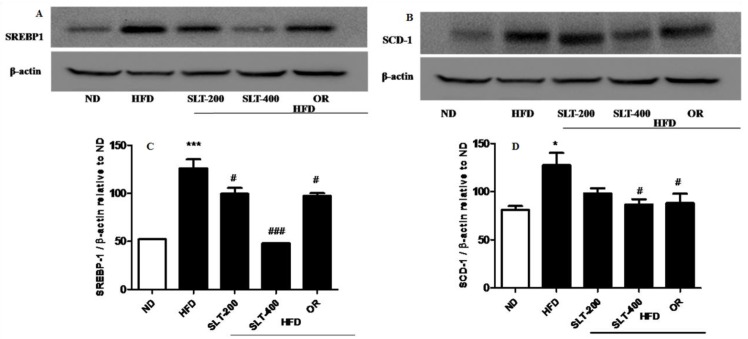
SLT supplementation decreased hepatic lipogenic key protein expression in HFD mice. Representative blotting of lipogenic proteins (**A**) SREBP-1 and (**B**) SCD-1, and (**C**,**D**) summary of statistical data. Values given are mean ± SEM (*n* = 3–6). One-way analysis of variance (ANOVA) followed by Tukey’s test were used to compare all pairs of columns. *n* = 6, * *p* < 0.05, and *** *p* < 0.001 versus ND; ^#^ *p* < 0.05, and ^###^ *p* < 0.001 versus HFD.

To further explore the mechanism of SLT in the reduction of the blood lipid index and fatty liver formation induced by HFD, the mRNA expressions of lipogensis marker genes were examined. Our data showed that HFD supplementation significantly up-regulated the expression levels of *Lxr-α, Fasn, Acac-β, Srebf-1*, and *Scd1* compared to the ND group (Figure 5A–E). Moreover, compared with mice fed the HFD, the SLT or OR administration significantly decreased the expression level of these lipogensis marker genes. In addition, the administration of SLT or OR decreased the protein expression of SREBF1 and SCD-1, as evidenced by Western blotting analysis (Figure 6A,B). Furthermore, a higher dosage of SLT resulted in even higher reductions in the expression of lipogenesis genes and SREBF1 and SCD-1 proteins than did SLT-200 and OR administration (Figure 5 and Figure 6).

**Figure 7:**
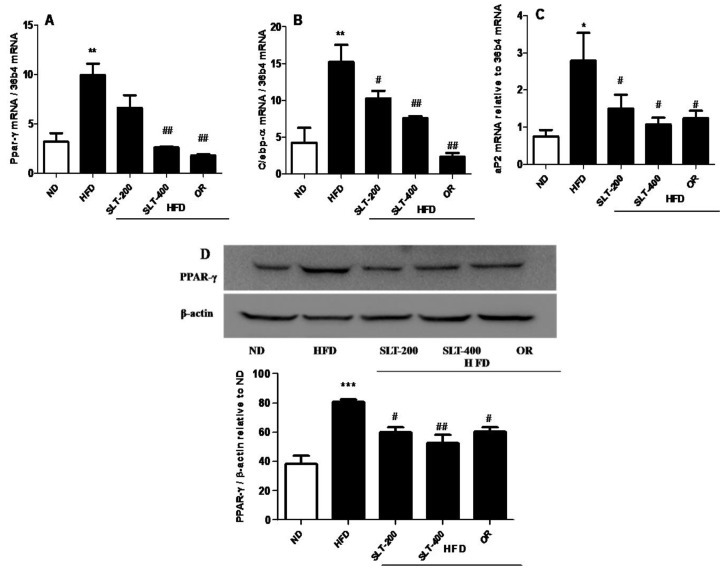
SLT administration prevented hepatic adipogenic gene and protein over-expression in HFD C57BL/6 mice. (**A**) PPAR-γ, (**B**) C/EPB-α, (**C**) aP2, and a representative figure of PPAR-γ blotting and a summary of the data (**D**). Values given are mean ± SEM (*n* = 3–6). One-way analysis of variance (ANOVA) followed by Tukey’s test were used to compare all pairs of columns. * *p* < 0.05, ** *p* < 0.01, and *** *p* < 0.001 versus ND. ^#^ *p* < 0.05, ^##^ *p* < 0.01 versus HFD.

In addition, the mRNA expression level of hepatic adipogenesis-related genes was also evaluated to examine the SLT-mediated reduction in adipogenesis. As shown in Figure 7, an intragastric administration of SLT or OR prevented the over-expression of *Ppar-γ* (A), *C/ebp-α* (B), and *aP2* (C) in the liver tissue of HFD mice. 

The Western blotting showed that supplementation of SLT or OR significantly prevented the elevation of PPAR-γ expression in the liver tissue (Figure 7D). Together, our results suggested that a higher dosage of SLT (400 mg/kg BW) supplementation significantly blocked the mRNA over-expression of adipogenic-related genes, which in turn attenuated the adipogenic-related protein PPAR-γ expression in the liver of HFD mice.

## 4. Discussion

A growing body of pharmacological studies has suggested the beneficial effects of *G. pentaphyllum*, *M. alba*, and *N. nucifera* on human health. SLT, a medical formula consisting of those three plants, has long been used to prevent obesity and related complications. We tentatively identified 24 metabolites by an untargeted metabolomic analysis of SLT, and most of them were flavonols and their derivatives. Five quercetin analogs shared fragment ions at *m/z* 303, while three kaempferol analogs shared fragment ions at *m/z* 284/285. These fragment ions and this fragmentation pattern of quercetin and kaempferol derivatives fit well with previous reports on flavonol derivatives in herbs that contain SLT [16,36]. Phytochemical and spectroscopic studies on herbs containing SLT suggested that quercetin and kaempferol derivatives were the most abundant metabolites [11,16,17,19]. These reports were consistent with our UHPLC Q-Orbitrap analysis data, which showed that analogs of quercetin and kaempferol were major compounds of flavonoids in SLT.

In the present study, we found that the dietary supplementation of SLT prevented obesity-related complications in HFD-fed mice. Mice administered 200 and 400 mg/kg BW of SLT showed a significant reduction in body weight gain. Prevention of body weight gain and fat storage is important in the treatment of obesity-related complications. Our data proved that SLT administration significantly prevented the body weight gain of long-term HFD-fed mice and also ameliorated the elevated serum total cholesterol, TG, LDL-C, ALT, and AST to near-normal levels. The prevention of weight gain and retention of serum biochemical parameters by a higher dosage of SLT administration can be correlated with the suppression of lipid synthesis in the hepatic tissue of HFD mice. It is worth noting that these preventive effects of high dosages of SLT were as good as positive OR administration.

Our data showed that impaired glucose tolerance and insulin sensitivity in the liver of HFD-fed mice were effectively improved by both a dose of SLT or OR. In vivo studies suggested that changes in the insulin sensitivity in the liver are responsible for the transcriptional regulation of lipogenesis genes, such as *Srebp-1c*, *Fas*, and *Scd* [37]. Numerous in vitro studies reported that obesity has been prevented by inhibiting hepatic lipogenesis as well as suppressing hepatic adipogenesis [10,38,39]. 

In order to understand the molecular mechanism of SLT in alleviating obesity, we performed real-time PCR to analyze the genes involved in the lipogenesis in the hepatic tissue. We observed that the HFD-induced expression of lipogenic genes, including *Lxr-α*, *Srebp1*, *Fas*, and *Scd1*, was effectively suppressed by a higher dosage of SLT. A previous investigation carried out in HFD-fed animals clearly suggested that quercetin supplementation altered the expression of diverse lipid metabolism-related genes in the hepatic tissue and reduced fat accumulation in the liver tissue of mice fed HFD [20]. Our data showing the inhibition of lipogenesis was further supported by histological observation, in which a higher dose of SLT supplementation caused minimal or no intracytoplasmic deposition of fat droplet distribution in the liver parenchymal cells (Figure 5B). Therefore, we propose that the inhibition of hepatic lipogenesis and the reduction in liver fat accumulation was caused by quercetin analogs, as indicated by the chemical profiling analysis of SLT. Furthermore, our Western blotting data revealed that the expression of SREBP-1 and SCD-1 were down-regulated by the higher dose of SLT supplementation. SREBP-1 is a receptor molecule that controls the enzymes involved in fatty acid oxidation and synthesis. However, SCD-1 is a key molecule, implicated in the process of acetyl-CoA to synthesize palmitate (C16:0), and forms a double bond in stearoyl-CoA. A recent study has evidently reported that the supplementation of the herbal extract of *Valeriana dageletiana* lowered the expression of genes involved in lipogenesis such as *Srebp1*, *Fas,* and *Scd1* [21].

SLT administration did not alter the epidermal white adipose tissue mass, while liver weight gain and enlargement were significantly prevented by SLT when compared to the mice fed the HFD alone. Our finding was supported by a previous report, which indicated that treatment with herbal extract of *Solidago virgaurea* did not show a significant influence on the adipose tissue weight in high-fat-diet-induced obese mice, while the lipogenesis and adipogenesis in the hepatic tissues was effectively prevented [10]. Our real-time PCR analysis showed that the supplementation of SLT significantly prevented the over-expression of hepatic adipogenic genes such as *Ppar-γ*, *C/ebp-α*, and *aP2*, and the protein expression of PPAR-γ in the liver tissue was also blocked by SLT-treated HFD mice. PPAR-γ is an important transcriptional factor and is normally expressed in very low levels in liver. Abnormalities in the liver, including adipogenic hepatic steatosis, induce the over-expression of this transcription factor and consequently induce the over-expression of several adipogenic genes, such as *aP2* and *C/ebp-α*, in the hepatic tissue. Moreover, several studies found that PPAR-γ has an important role in hepatic steatosis and adipogenesis in the liver [38,39,40]. PPAR-γ and C/EBP-α, which are involved in the self-regulation loop, mediate the expression of lipid metabolizing enzymes FAS and SCD-1, as well as aP2, which is a fatty acid carrier protein. Several in vitro studies also reported that the disruption of hepatic PPAR-γ expression led to insulin resistance and hepatic steatosis [39].

Previous research found that kaempferol and quercetin abundant extracts from *Hippophae rhamnoides* seeds reduced the expression of hepatic *Ppar-γ*, *C/ebp-α*, and *aP2* in obese mice [41]. Further, Lee at al. also reported that kaempferol isolated from *N. nucifera*, one of the herbs contained in SLT, reduced the expression of different adipogenic transcription factors and their target genes [14]. Our results have been well supported by these reports. Therefore, we propose here that kaempferol and quercetin derivatives of SLT could be the principle bioactive metabolites suppressing the hepatic adipogenesis and steatosis in HFD mice.

Our results imply that the prevention of fatty liver formation by SLT in HFD C57BL/6 mice may be attributed to the inhibition of the lipogenic and adipogenic pathways, indicated by metabolic enzymes in the liver but not in the lipid oxidative pathway. This result is evidenced by our previous report that Big Yellow Tea prevented steatosis by inhibiting lipid synthesis, but not the lipid oxidation pathway, in db/db mice [42]. Chen et al. also reported that kefir improved fatty liver syndrome by inhibiting the lipogenesis pathway in ob/ob mice [43]. These data indicated that the lipogenesis pathway might be more important than lipid oxidation for fatty liver syndrome. In addition, our data showed that SLT treatment had almost the same effect as Orlistat in preventing body weight gain in HFD mice. Orlistat is a gastric and pancreatic lipase inhibitor that reduces dietary fat absorption [44,45]. Therefore, it is possible that SLT supplementation might also inhibit intestinal fat absorption, thus preventing body weight gain in HFD mice.

**Figure 8:**
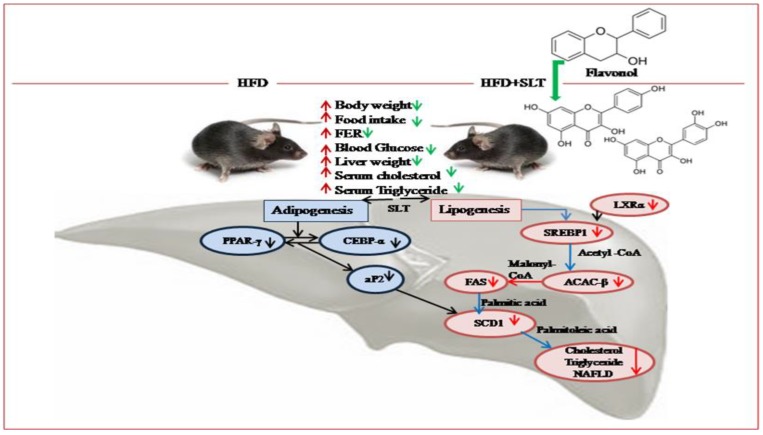
Proposed mechanism of SLT-mediated modulation on the lipid metabolism pathway and hepatic non-alcoholic fatty liver disease (NAFLD) in C57BL/6 mice. The significant effect, beneficial to health, of SLT on the prevention of fatty liver formation may occur through the inhibition of metabolic enzymes of the lipogenic and adipogenic pathway, but not of the lipid oxidative pathway.

Based on our data, we propose a mechanism of SLT modulation on the lipid metabolism pathway and hepatic NAFLD in C57BL/6 mice (Figure 8). The inhibitory mechanism of SLT on lipid metabolism was achieved by repressing the fatty acid transport and storage via the suppression of signaling pathways that mediate lipogenesis along with adipogenesis in HFD-induced C57BL/6 mice. In addition, our metabolomic data suggested that flavonol derivatives rich in SLT might be functional compounds for attenuating HFD-induced NAFLD and obesity complications in C57BL/6 mice.

## 5. Conclusions

The administration of SLT prevented body weight gain, suppressed elevated blood glucose, serum cholesterol, and triglyceride, and reduced hepatic lipid accumulation as well as liver weight gain in HFD-induced C57BL/6 mice. Moreover, SLT supplementation suppressed lipogenesis and adipogenesis genes, as well as the expression of key proteins involved in lipogenic mechanism, in the livers of mice. The preventive effects of SLT against obesity complications might be attributed to the synergetic effect of flavonol derivatives such as quercetin and kaempferol analogs. Taken together, the SLT formula might be the ideal combination of a potential health drink for preventing NAFLD and obesity complications.

## Figures and Tables

**Table 1:** Primer sequences designed for mice genes.

Gene Name	Primer Forward (5′–3′)	Primer Reverse (5′–3′)
*36b4*	CCCTGAAGTGCTCGACATCA	TGCGGACACCCTCCAGAA
*aP2*	AACACCGAGATTTCCTTCAA	TCACGCCTTTCATAACACAT
*Acac-β*	AGACACTGCAAATCCCAACCTTAC	CTTCGTCCACATCCTTCACACA
*Cebp-α*	AGACATCAAGCGCCTACATCG	TGTAGGTGCATGGTGGTCTG
*Fasn*	CGTGTGACCGCCATCTATATCG	TGAGGTTGCTGTCGTCTGTAGTCTT
*Lxr-α*	CGACAGAGCTTCGTCCACAA	CGTTCCCCAGCATTTTGG
*Ppar-* *γ*	GAAAGACAACGGACAAATCACCAT	CGGCTTCTACGGATCGAAACTG
*Scd1*	TCCTCCTTGGATTGTGTAGAAACTT	AATGTCAGAAGAAATCAGGTGGGTA
*Srebf1*	AGTCCAGCCTTTGAGGATAGCC	CCGTAGCATCAGAGGGAGTGAG

**Table 2:** Ultrahigh-performance liquid chromatography (UHPLC)-quadrupole-orbitrap high-resolution mass spectrometry parameters of the 24 Sanglan Tea metabolites.

ID	t_R_ (min)	^a^ MS (M + H)^+^/(M − H)^−^	UPLC-ESI MS Ion Fragments (*m*/*z*)	Predicted Formula	Assigned Identification	Substance class
1	3.47	272.12/270.11	256, 273, 226, 162, 155, 108/162	C_16_H_17_NO_3_	Norcoclaurine	Benzylisoquinoline alkaloid
2	3.77	611.15/609.14	303, 85/300, 271	C_26_H_26_ O_17_	Quercetin-3-*O*-arabinose-glucuronide	Flavonoid
3	3.77	613.54/610.52	303/301, 300	C_27_H_32_O_16_	Quercetin-3-*O*-rhamnoside-glucoside	Flavonoid
4	3.85	479.08/477.06	303/301	C_21_H_18_O_13_	Quercetin-3-*O*-glucuronide	Flavonoid
5	3.85	465.10/463.08	303/301	C_21_H_20_O_12_	Quercetin-3-*O*-galactoside	Flavonoid
6	3.85	303.18/301.03	303.18, 237.10, 171.01/301	C_15_H_10_O_7_	Quercetin	Flavonoid
		**^b^** MS (M + H)^+^				
7	0.87	148.98	148.98, 130.97, 1125.96	C_5_H_9_NO_4_	Glutamine	Amino acid
8	1.37	276.14	259, 230	C_17_H_9_NO_3_	Liriodenine	Aporphine alkaloid
9	3.69	611.31	611.31, 489.24, 206.12	C_27_H_30_O_16_	Rutin	Flavonoid
10	3.77	328.15	329, 314, 313, 298,	C_20_H_25_NO_3_	*O*-Methylarmepavine	Benzylisoquinoline alkaloid
11	3.82	314.17	314, 283, 268	C_19_H_24_NO_3_	Lotusine	Benzylisoquinoline alkaloid
12	4.17	266.11	249,219	C_17_H_15_NO_2_	Anonaine	Aporphine alkaloid
13	4.23	296.16	265, 250, 235	C_19_H_21_NO_2_	Nuciferine	Aporphine alkaloid
14	5.44	331.19	331.19	C_17_H_14_O_7_	Rhamnazin	Flavonoid
		^c^ MS (M − H)				
15	3.50	577.13	289, 175	C_30_H_26_O_12_	Procyanidin B	Flavonoid
16	3.72	353.19	191, 173, 179	C_16_H_18_O_9_	4-Caffeoylquinic acid	Cryptochlorogenic acid
17	3.75	595.13	301	C_26_H_28_O_16_	Quercetin-3-*O*-arabinose-galactoside	Flavonoid
18	3.77	609.14	300, 271, 255, 301	C27H30O16	Quercetinrutinoside isomer	Flavonoid
19	3.77	607.50	300, 284, 271, 255	C27H28O16	Kaempferol-3-*O*-rhamnoside-galacturonide	Flavonoid
20	3.78	133.01	115, 87, 71	C_4_H_6_O_5_	L-Malic acid	Hydroxy acids and derivatives
21	3.82	593.15	284, 255, 227	C_27_H_30_O_15_	Kaempferol-3-*O*-rhamnopyranosyl-(1→6)-glucopyranoside	Flavonoid
22	3.85	463.08	301	C_21_H_20_O_12_	Hirsutrin	Flavonoid
23	3.85	463.08	301, 300, 271, 256, 255	C_21_H_20_O_12_	Quercetin-3-*O*-glucopyranoside (isoquercetin)	Flavonoid
24	3.93	447.13	285, 257, 255, 229, 245, 227	C_21_H_20_O_11_	Kaempferol 3-*O*-glucoside (astragalin)	Flavonoid

^a^ Compounds identified in both positive and negative ion mode; ^b^ Compounds identified in positive ion mode; ^c^ Compounds identified in negative ion mode.

**Table 3:** Description of the metabolic parameters measured in the study.

GROUPS	ND	HFD	HFD + SLT 200	HFD + SLT 400	HFD + OR
Body weight gain (g)	11.05 ± 2.20 ^a^	34.25 ± 2.41 ^b^	25.2 ± 3.56 ^c^	19.52 ± 3.30 ^d^	23.14 ± 2.043 ^c^
Food intake (Kcal/day)	12.6 ± 0.03 ^a^	15.0 ± 0.08 ^b^	14.04 ± 0.1 ^b^	14.56 ± 0.2 ^b^	14.56 ± 0.12 ^b^
FER	3.15 ± 0.72 ^a^	11.81 ± 0.86 ^b^	9.33 ± 2.43 ^c^	6.97 ± 3.01 ^d^	8.26 ± 1.67 ^c^
Fasting Glucose (mmol L^−1^)	7.6 ± 0.93 ^a^	13.4 ± 1.42 ^b^	9.8 ± 0.91 ^c^	9.2 ± 0.86 ^c^	10.1 ± 1.04 ^c^
Subcutaneous fat mass relative to BW (%)	2.4 ± 0.55 ^a^	3.9 ± 0.80 ^b^	4.0 ± 0.61 ^b^	4.1 ± 0.54 ^b^	4.1 ± 0.56 ^b^
Liver weight	1.14 ± 0.13 ^a^	3.31 ± 0.47 ^b^	1.94 ± 0.31 ^a^	1.56 ± 0.26 ^a^	1.76 ± 0.42 ^a^
Liver weight relative to BW (%)	3.6 ± 0.48 ^a^	5.9 ± 0.58 ^b^	3.8 ± 0.47 ^a^	3.6 ± 0.80 ^a^	3.9 ± 0.62 ^a^

Abbreviations: ND: normal diet; HFD: high-fat diet; SLT-200: Sanglan tea 200 mg/kg BW; SLT-400: Sanglan Tea 400 mg/kg BW; OR: Orlistat 10 mg/kg BW. FER = Body weight gain (g/day)/Food intake (g/day). Data are presented as mean ± SEM (*n* = 10–12). Values in a row with different letters are significantly (*p* < 0.05) different from each other.

## Supplementary Materials

The followings are available online at http://www.mdpi.com/2072-6643/10/9/1276/s1, Figure S1: Total ion chromatogram-summing up intensities of all mass spectral peaks of SLT by UPLC-Q-Orbitrap; TIC(+POS)-Positive ion mode, Figure S2: Total ion chromatogram-summing up intensities of all mass spectral peaks of SLT by UPLC-Q-Orbitrap; TIC(-NEG)-Negative ion mode.

## References

[1] Hotamisligil G.S. (2006). Inflammation and metabolic disorders. Nature.

[2] Zobel E.H., Hansen T.W., Rossing P., von Scholten B.J. (2016). Global Changes in Food Supply and the Obesity Epidemic. Curr. Obes. Rep..

[3] Rodríguez-Monforte M., Sánchez E., Barrio F., Costa B., Flores-Mateo G. (2017). Metabolic syndrome and dietary patterns: A systemic review and analysis of observational studies. Eur. J. Nutr..

[4] Hussain A., Yadav M.K., Bose S., Wang J.H., Lim D., Song Y.K., Ko S.G., Kim H. (2016). Daesiho-Tang Is an Effective Herbal Formulation in Attenuation of Obesity in Mice through Alteration of Gene Expression and Modulation of Intestinal Microbiota. PLoS ONE.

[5] Razmovski-Naumovski V., Huang T.H.W., Tran V.H., Li G.Q., Duke C.C. (2005). Chemistry and pharmacology of *Gynostemma pentaphyllum*. Phytochem. Rev..

[6] Yang Y., Yang X., Xu B., Zeng G., Tan J., He X., Hu C., Zhou Y. (2014). Chemical constituents of *Morus alba* L. and their inhibitory effect on 3T3-L1 preadipocyte proliferation and differentiation. Fitoterapia.

[7] Ma C., Li G., He Y., Xu B., Mi X., Wang H., Wang Z. (2015). Pronuciferine and nuciferine inhibit lipogenesis in 3T3-L1 adipocytes by activating the AMPK signaling pathway. Life Sci..

[8] Jang H., Lee J.W., Lee C., Jin Q., Lee M.K., Lee C.K., Lee M.K., Hwang B.Y. (2016). Flavonol glycosides from the aerial parts of *Gynostemma pentaphyllum* and their antioxidant activity. Arch. Pharm. Res..

[9] Huang T.H., Tran V.H., Roufogalis B.D., Li Y. (2007). Gypenoside XLIX, a naturally occurring PPAR-alpha activator, inhibits cytokine-induced vascular cell adhesion molecule-1 expression and activity in human endothelial cells. Eur. J. Clin. Pharmacol..

[10] Wang Z., Kim J.H., Jang Y.S., Kim C.H., Lee J.Y., Lim S.S. (2017). Anti-obesity effect of *Solidago virgaurea var. gigantea* extract through regulation of adipogenesis and lipogenesis pathways in high-fat diet-induced obese mice (C57BL/6N). Food Nutr. Res..

[11] Hong M., Cai Z., Song L., Liu Y., Wang Q., Feng X. (2018). *Gynostemma pentaphyllum* attenuates the progression of nonalcoholic fatty liver disease in mice: A biomedical investigation integrated with in silico assay. Evid. Based Complement. Altern. Med..

[12] Xing S.F., Liu L.H., Zu M.L., Ding X.F., Cui W.Y., Chang T., Piao X.L. (2018). the inhibitory effect of gypenoside stereoisomers, gypenoside L and gypenoside LI, isolated from *Gynostemma pentaphyllum* on the growth of human lung cancer A549 cells. J. Ethnopharmacol..

[13] Zhang H., Chen X., Zong B., Yuan H., Wang Z., Wei Y., Wang X., Liu G., Zhang J., Li S. (2018). Gypenosides improve diabetic cardiomyopathy by inhibiting ROS-mediated NLRP3 inflammasome activation. J. Cell. Mol. Med..

[14] Lee B., Kwon M., Choi J.S., Jeong H.O., Chung H.Y., Kim H.R. (2015). Kaempferol isolated from *Nelumbo nucifera* inhibits lipid accumulation and increases fatty acid oxidation signaling in adipocytes. J. Med. Food.

[15] Zhang C., Deng J., Liu D., Tuo X., Xiao L., Lai B., Yao Q., Liu J., Yang H., Wang N. (2018). Nuciferine ameliorates hepatic steatosis in high-fat diet/streptozocin-induced diabetic mice through PPARα/PGC1α pathway. Br. J. Pharmacol..

[16] Mukherjee P.K., Mukherjee D., Maji A.K., Rai S., Heinrich M. (2009). The sacred lotus (*Nelumbo nucifera*)-phytochemical and therapeutic profile. J. Pharm. Pharmacol..

[17] Sánchez-Salcedo E.M., Tassotti M., Del Rio D., Hernández F., Martínez J.J., Mena P. (2016). (Poly) phenolic fingerprint and chemometric analysis of white (*Morus alba* L.) and black (*Morus nigra* L.) mulberry leaves by using a non-targeted UHPLC–MS approach. Food Chem..

[18] Hunyadi A., Liktor-Busa E., Márki A., Martins A., Jedlinszki N., Hsieh T.J., Bathori M., Hohmann J., Zupko I. (2013). Metabolic effects of mulberry leaves: Exploring potential benefits in type 2 diabetes and hyperuricemia. Evid. Based Complement. Altern. Med..

[19] Liu S., Li D., Huang B., Chen Y.X., Lu X.C., Wang Y.W. (2013). Inhibition of pancreatic lipase, -glucosidase, -amylase, and hypolipidemic effects of the total flavonoids from *Nelumbo nucifera* leaves. J. Ethnopharmacol..

[20] Jung C.H., Cho I., Ahn J., Jeon T.I., Ha T.Y. (2013). Quercetin reduces high-fat diet-induced fat accumulation in the liver by regulating lipid metabolism genes. Phytother. Res..

[21] Wang Z., Hwang S.H., Kim J.H., Lim S.S. (2017). Anti-Obesity Effect of the Above-Ground Part of *Valeriana dageletiana* Nakai ex F. Maek Extract in High-Fat Diet-Induced Obese C57BL/6N Mice. Nutrients.

[22] Malek M.A., Hoang M.H., Jia Y., Lee J.H., Jun H.J., Lee D.H., Lee H.J., Lee C., Lee M.K., Hwang B.Y. (2013). Ombuin-3-O-β-D-glucopyranoside from *Gynostemma pentaphyllum* is a dual agonistic ligand of peroxisome proliferator-activated receptors α and δ/β. Biochem. Biophys. Res. Commun..

[23] Jeszka-Skowron M., Flaczyk E., Jeszka J., Krejpcio Z., Król E., Buchowski M.S. (2014). Mulberry leaf extract intake reduces hyperglycaemia in streptozotocin (STZ)-induced diabetic rats fed high-fat diet. J. Funct. Foods.

[24] Ono Y., Hattori E., Fukaya Y., Imai S., Ohizumi Y. (2006). Anti-obesity effect of *Nelumbo nucifera* leaves extract in mice and rats. J. Ethnopharmacol..

[25] Horžić D., Jambrak A.R., Belščak-Cvitanović A., Draženka K., Vesna L. (2012). Comparison of Conventional and Ultrasound Assisted Extraction Techniques of Yellow Tea and Bioactive Composition of Obtained Extracts. Food Bioprocess Technol..

[26] Tian L., Zeng K., Shao W., Yang B.B., Fantus I.G., Weng J., Jin T. (2015). Short-Term Curcumin Gavage Sensitizes Insulin Signaling in Dexamethasone-Treated C57BL/6 mice. J. Nutr..

[27] Cho J., Koh Y., Han J., Kim D., Kim T., Kang H. (2016). Adiponectin mediates the additive effects of combining daily exercise with caloric restriction for treatment of non-alcoholic fatty liver. Int. J. Obes..

[28] Xie Z., Gong M.C., Su W., Turk J., Guo Z. (2007). Group VIA phospholipase A_2_ (iPLA_2_β) participates in angiotensin II-induced transcriptional up-regulation of regulator of G-protein signaling-2 in vascular smooth muscle cells. J. Biol. Chem..

[29] Xie Z., Liu D., Liu S., Calderon L., Zhao G., Turk J., Guo Z. (2011). Identification of a cAMP-response element in the regulator of G-protein signaling-2 (RGS2) promoter as a key Cis-regulatory element for RGS2 transcriptional regulation by angiontensin II in cultured vascular smooth muscles. J. Biol. Chem..

[30] Xie Z., Su W., Guo Z., Pang H., Post S.R., Gong M.C. (2006). Up-regulation of CPI-17 phosphorylation in diabetic vasculature and high glucose cultured vascular smooth muscle cells. Cardiovasc. Res..

[31] Xie Z., Su W., Liu S., Zhao G., Esser K., Schroder E.A., Lefta M., Stauss H.M., Guo Z., Gong M.C. (2015). Smooth-muscle BMAL1 participates in blood pressure circadian rhythm regulation. J. Clin. Investig..

[32] HMDB (The Human Metabolome Database). http://www.hmdb.ca/.

[33] GNPS (The Global Natural Product Social Molecular Networking). http://gnps.ucsd.edu.

[34] TCMSP (Traditional Chinese Medicine Systems Pharmacology and analysis platform. http://sm.nwsuaf.edu.cn/lsp/tcmsp.php.

[35] Jiménez-López J., Ruiz-Medina A., Ortega-Barrales P., Llorent-Martínez E.J. (2017). Phytochemical profile and antioxidant activity of caper berries (*Capparis spinosa* L.): Evaluation of the influence of the fermentation process. Food Chem..

[36] Guo Y., Chen X., Qi J., Yu B. (2016). Simultaneous qualitative and quantitative analysis of flavonoids and alkaloids from the leaves of *Nelumbo nucifera* Gaertn. using high-performanceliquid chromatography with quadrupole time-of-flight mass spectrometry. J. Sep. Sci..

[37] Sanders F.W., Griffin J.L. (2016). De novo lipogenesis in the liver in health and disease: More than just a shunting yard for glucose. Biol. Rev. Camb. Philos. Soc..

[38] Evans R.M., Barish G.D., Wang Y.X. (2004). PPARs and the complex journey to obesity. Nat. Med..

[39] Matsusue K., Haluzik M., Lambert G., Yim S.H., Gavrilova O., Ward J.M., Brewer B. Jr., Reitman M.L., Gonzalez F.J. (2003). Liver-specific disruption of PPAR_in leptin-deficient mice improves fatty liver but aggravates diabetic phenotypes. J. Clin. Investig..

[40] Yu S., Matsusue K., Kashireddy P., Cao W.Q., Yeldandi V., Yeldandi A.V., Rao M.S., Gonzalez F.J., Reddy J.K. (2003). Adipocyte-specific gene expression and adipogenic steatosis in the mouse liver due to peroxisome proliferator-activated receptor -1 (PPAR-1) over expression. J. Biol. Chem..

[41] Yang X., Wang Q., Pang Z.R., Pan M.R., Zhang W. (2017). Flavonoid-enriched extract from *Hippophae rhamnoides* seed reduces high fat diet induced obesity, hypertriglyceridemia, and hepatic triglyceride accumulation in C57BL/6 Mice. Pharm. Biol..

[42] Ten Y., Li D., Guruvaiah P., Xu N., Xie Z. (2018). Dietary Supplement of Large Yellow Tea Ameliorates Metabolic Syndrome and Attenuates Hepatic Steatosis in db/db Mice. Nutrients.

[43] Chen H.L., Tung Y.T., Tsai C.L., Lai C.W., Lai Z.L., Tsai H.C., Lin Y.L., Wang C.H., Chen C.M. (2014). Kefir improves fatty liver syndrome by inhibiting the lipogenesis pathway in leptin-deficient ob/ob knockout mice. Int. J. Obes..

[44] Hauptman J. (2000). Orlistat: Selective inhibition of caloric absorption can affect long-term body weight. Endocrine.

[45] Drew B.S., Dixon A.F., Dixon J.B. (2007). Obesity management: Update on orlistat. Vasc. Health Risk Manag..

