# Supplementary material for: Preventive Effect of Flavonol Derivatives Abundant Sanglan Tea on Long-Term High-Fat-Diet-Induced Obesity Complications in C57BL/6 Mice
Source: Nutrients. 2018 Sep 10;10(9):1276. doi: 10.3390/nu10091276 (PMC6164069; doi:10.3390/nu10091276)
Supplement: Supplementary file 1 [file nutrients-10-01276-s001.pdf]

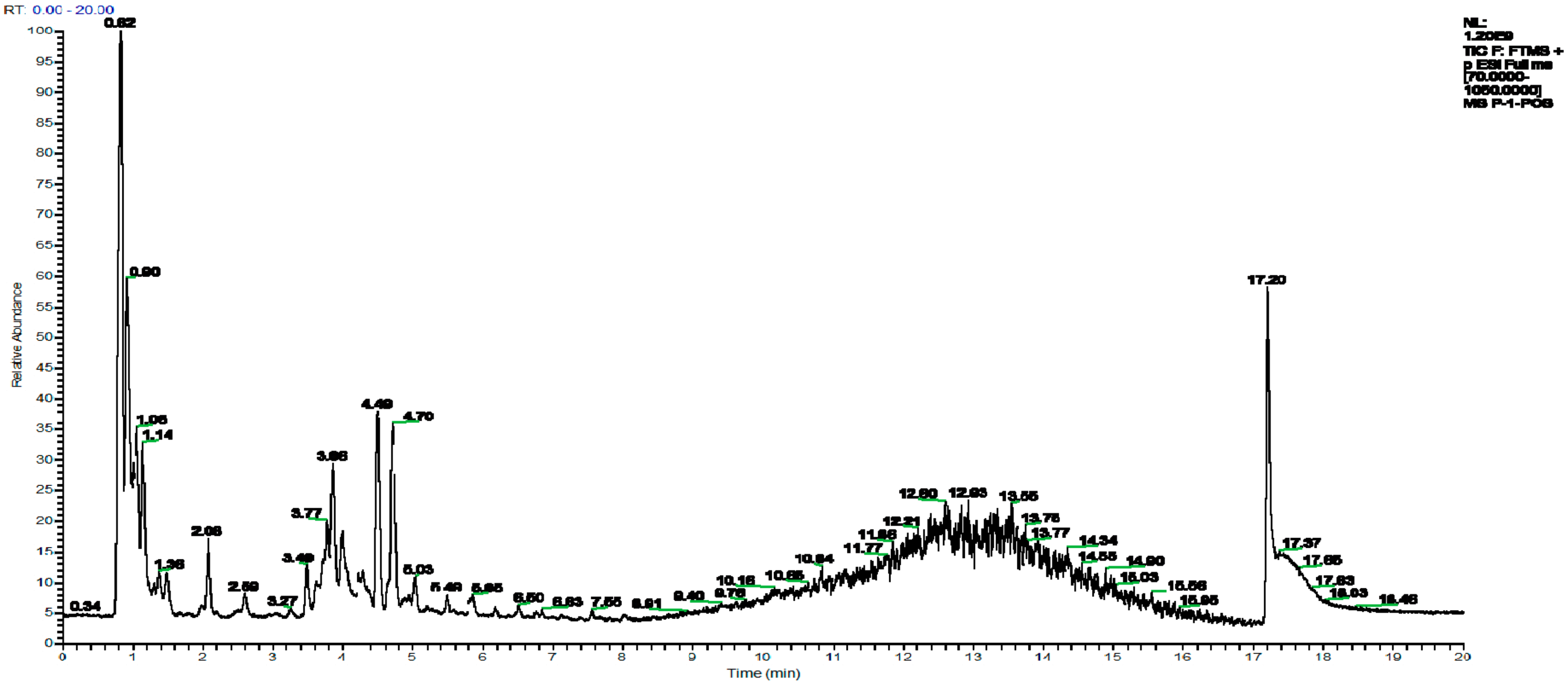

Supplementary Figure 1-Total ion chromatogram-summing up intensities of all mass spectral peaks of SLT by UPLC-Q-Orbitrap; TIC(+POS)-Positive ion mode.

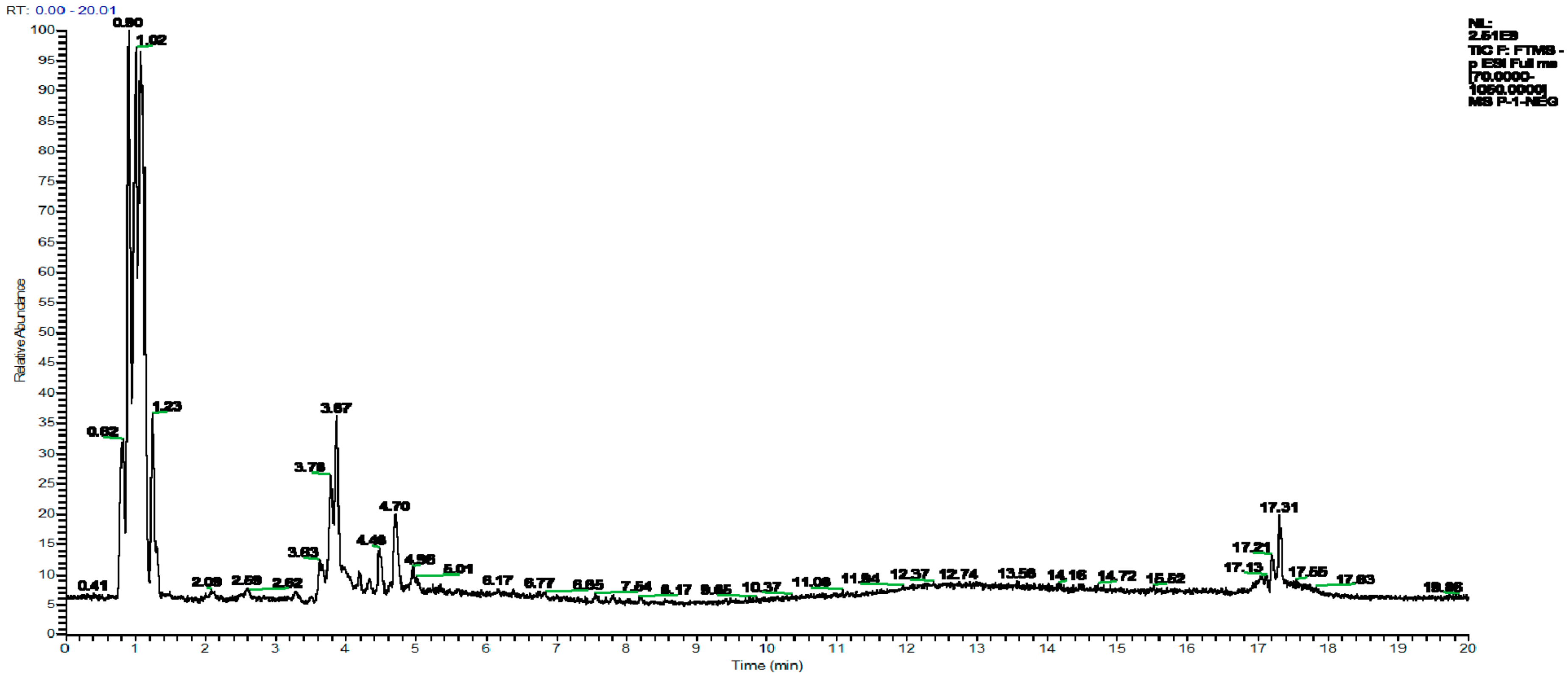

Supplementary Figure 2-Total ion chromatogram-summing up intensities of all mass spectral peaks of SLT by UPLC-Q-Orbitrap; TIC(-NEG)-Negative ion mode.
